# LPGAT1 controls the stearate/palmitate ratio of phosphatidylethanolamine and phosphatidylcholine in sn-1 specific remodeling

**DOI:** 10.1016/j.jbc.2022.101685

**Published:** 2022-02-04

**Authors:** Yang Xu, Paighton C. Miller, Colin K.L. Phoon, Mindong Ren, Titli Nargis, Sujith Rajan, M. Mahmood Hussain, Michael Schlame

**Affiliations:** 1 Department of Anesthesiology, New York University Grossman School of Medicine, New York, New York, USA; 2 Department of Pediatrics, New York University Grossman School of Medicine, New York, New York, USA; 3 Department of Cell Biology, New York University Grossman School of Medicine, New York, New York, USA; 4 Department of Foundations of Medicine, New York University Long Island School of Medicine, Mineola, New York, USA

**Keywords:** acyltransferase, metabolism, molecular species, obesity, phospholipid, CL, cardiolipin, dMePE, dimethylphosphatidylethanolamine, FBS, fetal bovine serum, LC-ESI-MS/MS, liquid chromatography electrospray-ionization tandem mass spectrometry, LPC, lysophosphatidylcholine, LPE, lysophosphatidylethanolamine, LPG, lysophosphatidylglycerol, LPGAT1, lysophosphatidylglycerol acyltransferase 1, MG, monoglyceride, PA, phosphatidic acid, PC, phosphatidylcholine, PE, phosphatidylethanolamine, PEMT, PE methyltransferase, PG, phospatidylglycerol, PI, phosphatidylinositol, TG, triglyceride

## Abstract

Most mammalian phospholipids contain a saturated fatty acid at the sn-1 carbon atom and an unsaturated fatty acid at the sn-2 carbon atom of the glycerol backbone group. While the sn-2 linked chains undergo extensive remodeling by deacylation and reacylation (Lands cycle), it is not known how the composition of saturated fatty acids is controlled at the sn-1 position. Here, we demonstrate that lysophosphatidylglycerol acyltransferase 1 (LPGAT1) is an sn-1 specific acyltransferase that controls the stearate/palmitate ratio of phosphatidylethanolamine (PE) and phosphatidylcholine. Bacterially expressed murine LPGAT1 transferred saturated acyl-CoAs specifically into the sn-1 position of lysophosphatidylethanolamine (LPE) rather than lysophosphatidylglycerol and preferred stearoyl-CoA over palmitoyl-CoA as the substrate. In addition, genetic ablation of LPGAT1 in mice abolished 1-LPE:stearoyl-CoA acyltransferase activity and caused a shift from stearate to palmitate species in PE, dimethyl-PE, and phosphatidylcholine. Lysophosphatidylglycerol acyltransferase 1 KO mice were leaner and had a shorter life span than their littermate controls. Finally, we show that total lipid synthesis was reduced in isolated hepatocytes of LPGAT1 knockout mice. Thus, we conclude that LPGAT1 is an sn-1 specific LPE acyltransferase that controls the stearate/palmitate homeostasis of PE and the metabolites of the PE methylation pathway and that LPGAT1 plays a central role in the regulation of lipid biosynthesis with implications for body fat content and longevity.

Phospholipids contain two fatty acids attached to the sn-1 and sn-2 carbon atoms of the glycerol group. In general, the sn-1 position is occupied by saturated fatty acids, primarily palmitate (C16:0) or stearate (C18:0). In contrast, the sn-2 position contains predominantly unsaturated fatty acids, the composition of which is regulated by fatty acid remodeling. The canonical remodeling pathway, widely known as the Lands cycle, starts with the removal of the sn-2 linked fatty acid (phospholipase A2) and is completed by the transfer of another fatty acid from acyl-CoA (acyltransferase). The cycle balances the composition of unsaturated fatty acids in the sn-2 position (1), which is thought to be critical for the physical properties, the metabolic fate, and the cellular function of phospholipids (2, 3). Several acyltransferases have been identified and shown to have distinct phospholipid head group and acyl specificities, confirming the importance of acyltransferases for the phospholipid species composition (4).

In addition to the Lands cycle, a separate pathway has been postulated for sn-1 linked fatty acids (5, 6). This pathway requires the combined action of a phospholipase A1 and an sn-1 specific acyltransferase. The idea of sn-1 remodeling has been tentatively supported by the discovery of an acyltransferase that incorporates stearic acid into the sn-1 position of phosphatidylinositol (PI) (7, 8, 9, 10) and by the data, suggesting the active reacylation of partially oxidized 1-lyso-phospholipids (11). However, sn-1 remodeling is not nearly as well established as sn-2 remodeling. In particular, it is not known whether sn-1 remodeling is a widespread phenomenon among phospholipids and whether it has functional significance.

Here, we establish lysophosphatidylglycerol acyltransferase 1 (LPGAT1) as a pivotal sn-1 remodeling enzyme and show that it is of central importance for the regulation of lipid metabolism. Lysophosphatidylglycerol acyltransferase 1 was first identified as acyltransferase based on sequence homology and expressed in insect cells, which suggested that lysophosphatidylglycerol (LPG) and stearoyl-CoA are the preferred substrates. This led to the assumption that LPGAT1 remodels phosphatidylglycerol (PG), an intermediate on the cardiolipin (CL) pathway, hence the name LPG acyltransferase 1 (12). However, a number of contradictions have emerged. First, LPGAT1 is located in the endoplasmic reticulum (12) whereas PG is formed and converted to CL in the inner mitochondrial membrane (13). Second, the preference for saturated acyl-CoA’s of LPGAT1 (12) does not match the species composition of either PG or CL (14). Finally, a germline LPGAT1 KO mouse model was recently created. In this model, deletion of LPGAT1 caused only small changes in the species composition of PG and CL but much larger changes in other phospholipids (14). Together, these data raise doubts as to whether LPGAT1 is indeed involved in the remodeling of PG.

What is further complicating the situation is that several studies have suggested a connection between LPGAT1 and triacylglycerol (TG) metabolism. Lysophosphatidylglycerol acyltransferase 1 is regulated by the same micro-RNA that controls the microsomal TG transfer protein (15) and variants in the LPGAT1 gene region are associated with obesity in Pima Indians (16). Knockout and knockdown of LPGAT1 in mice lowered the concentration of serum lipids and increased the fat content of liver (14, 17). Lysophosphatidylglycerol acyltransferase 1 was found to have monoacylglycerol (MG) acyltransferase activity, which in theory could explain the link to TG metabolism (17). However, it is difficult to conceptualize that LPGAT1 reacts with MG and LPG, two structurally very different substrates, unless it is devoid of any substrate specificity. Therefore, the data remain incomplete until the MG acyltransferase activity of LPGAT1 is rigorously compared to its activities with other substrates.

In summary, the true function of LPGAT1 remains obscure. It is clear that LPGAT1 is homologous to acyltransferases and carries acyltransferase activity (12, 17) but the endogenous substrates of LPGAT1 have not been unambiguously identified. Several independent studies have suggested an involvement of LPGAT1 in TG metabolism (14, 15, 16, 17) but nothing is known about the underlying mechanism. Thus, we set out to clarify the biological function of LPGAT1 and discovered that it is an sn-1 specific acyltransferase involved in regulating the stearate/palmitate ratio.

## Results

### Lysophosphatidylglycerol acyltransferase 1 deletion alters the palmitate/stearate ratio of PE, dMePE, and PC

**Figure 1:**
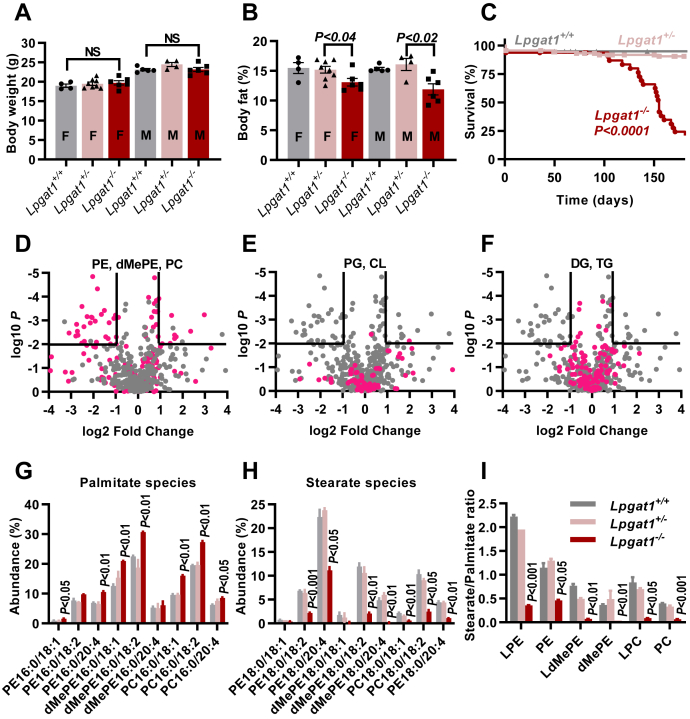
**Lysophosphatidylglycerol acyltransferase 1 deletion decreases the stearate/palmitate ratio of PE, dMePE, and PC.***A*, the body weight of 11 week old mice was measured. *B*, the body fat content of 11 week old mice was measured by the PIXImus small animal densitometer. *C*, survival was determined in *Lpgat1*^*+/+*^ (N = 40), *Lpgat1*^*+/−*^ (N = 90), and *Lpgat1*^*−/−*^ (N = 32) mice. Median survival of *Lpgat1*^*−/−*^ mice was 155 days. The survival curves of *Lpga1*^*+/+*^ and *Lpgat1*^*−/−*^ mice were compared by log-rank test. *D*–*F*, lipids were extracted from the livers of 5 months old mice and analyzed by LC-MS/MS. The lipidomes of *Lpgat1*^*+/+*^ and *Lpgat1*^*−/−*^ were compared by volcano plot. Molecular species of the indicated lipid classes are colored *red*. *G*–*I*, lipids were extracted from the livers of 5 months old mice and analyzed by LC-MS/MS. Abundancies represent the relative amount of the indicated species in the lipid class. The graphs show means ± SEM (N = 3). Comparisons between *Lpgat1*^*+/+*^ and *Lpgat1*^*−/−*^ were made by *t*-tests. CL, cardiolipin; DG, diglyceride; dMePE, dimethyl-PE; LPC, lysophosphatidylcholine; LPE, lysophosphatidylethanolamine; LdMePE, lysodimethylphosphatidylethanolamine; PC, phosphatidylcholine; PE, phosphatidylethanolamine; PG, phosphatidylglycerol; TG, triglyceride.

To identify functional deficits caused by LPGAT1 deletion, we bred *Lpgat1*^*−/−*^ mice from a heterozygous pair obtained through Jackson Laboratory. *Lpgat1*^*−/−*^ mice were born at the expected Mendelian ratio and developed without any apparent problem, reaching normal body weight at the age of 3 months (Fig. 1*A*). However, their body fat content was reduced by about 20% (Fig. 1*B*). A lean phenotype has already been reported in another LPGAT1 KO model but there it was associated with a slight reduction in body weight (14). Importantly, our *Lpgat1*^*−/−*^ mice exhibited a drastic increase in mortality during early adulthood, which reduced their average life span to about 5 months (Fig. 1*C*). Besides that, we observed high preweaning mortality in litters born to primigravida *Lpgat1*^*−/−*^ mice. Lack of milk spots in the newborns suggested that *Lpgat1*^*−/−*^ mothers were unable to nourish their litters, which was supported by successful rescue of the newborns when nursed by surrogate mothers. The high preweaning mortality disappeared in subsequent pregnancies.

We determined the expression levels of LPGAT1 in human and mouse tissues and found that it is ubiquitously expressed, consistent with previously published data (12). In humans, the highest expression level was found in liver, whereas in mice the highest levels were found in brain and testis (Fig. S1). Since previous data suggested a role of LPGAT1 in liver metabolism (14, 15, 17), we determined the effect of LPGAT1 deletion on the hepatic lipidome, including the lipid class composition and the molecular species composition within each class. While the relative abundance of lipid classes did not change in response to LPGAT1 deletion (Fig. S2), we found discrete changes in the molecular species composition of several phospholipids (Table S1). To identify the most affected lipid species, we analyzed the data by volcano plot (Fig. S3). Out of 450 lipid species, 39 showed a highly significant response to LPGAT1 deletion (>2-fold change, *p* < 0.01). Of those, 16 belonged to phosphatidylethanolamine (PE), 15 belonged to phosphatidylcholine (PC), and three belonged to dimethyl-PE (dMePE) (Table S2). Thus, the majority (87%) of species strongly affected by LPGAT1 deletion were associated with the PE methylation pathway (Fig. 1*D*). In contrast, neither the molecular composition of PG and CL (Fig. 1*E*) nor the molecular composition of diglyceride and TG (Fig. 1*F*) changed significantly in response to LPGAT1 deletion.

The PE methylation pathway proceeds through three consecutive transmethylation steps, all catalyzed by PE methyltransferase (PEMT). The enzyme converts PE first into methyl-PE, then into dMePE, and finally into PC (18). We found that LPGAT1 deletion increased the abundance of 16:0/18:1, 16:0/18:2, and 16:0/20:4 species (Fig. 1*G*) and decreased the abundance of 18:0/18:1, 18:0/18:2, and 18:0/20:4 species (Fig. 1*H*) in lipids of the PE methylation pathway. Minor species of PE, dMePE, and PC followed the same trend, that is, a decrease in stearate and an increase in palmitate in response to LPGAT1 deletion (Table S1). As a result, the stearate/palmitate ratio decreased substantially in PE, dMePE, and PC and also in their corresponding lysophospholipids (Fig. 1*I*). In contrast, PG, CL, sphingomyelin, and TG remained unaffected and only small deviations from the normal species composition were seen in phosphatidic acid (PA), PI, phosphatidylserine, and diglyceride (Fig. S4). Other tissues, including intestine, kidney, and testis, showed a similar response to LPGAT1 deletion as the liver (Fig. S5).

In summary, deletion of LPGAT1 led to replacement of stearate species by palmitate species in phospholipids of the PE methylation pathway, including PE, dMePE, and PC. This alteration in the molecular species composition was found in multiple tissues and was associated with the signs of metabolic impairment, including low body fat, insufficient milk formation, and early mortality.

### Lysophosphatidylglycerol acyltransferase 1 is a stearoyl-selective 1-lyso-2-acyl-PE acyltransferase

**Figure 2:**
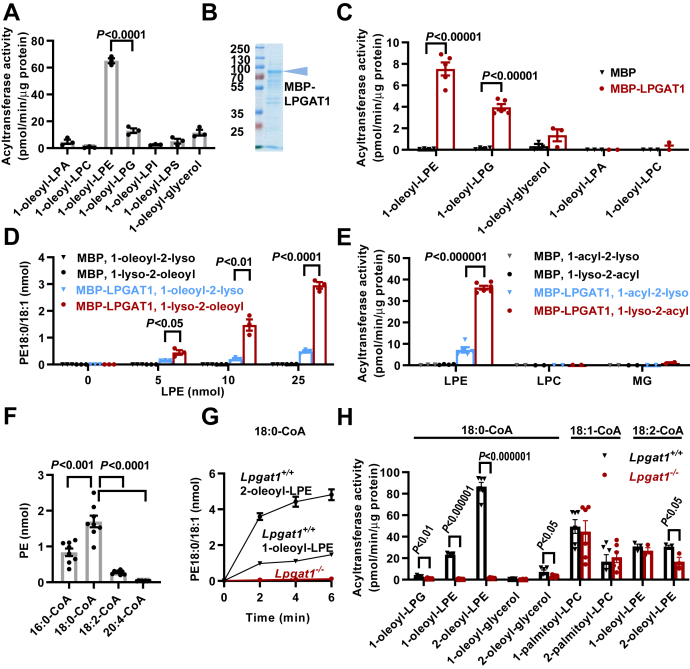
**Lysophosphatidylglycerol acyltransferase 1 is an sn-1 specific LPE acyltransferase.***A*, His-tagged murine LPGAT1 was expressed in Sf9 insect cells, solubilized in Triton X-100, and purified on Ni resin. Acyltransferase activities were measured in the purified enzyme with 1-oleoyl-2-lyso-lipids and stearoyl-CoA. *B*, MBP-tagged LPGAT1 was expressed in *E. coli*, solubilized with Triton X-100, purified on Amylose resin, and analyzed by SDS-PAGE with Coomassie staining. *C*–*E*, acyltransferase activities were measured in *E. coli* membranes expressing either MBP or MBP-LPGAT1. The substrates included the following: 1-oleoyl-2-lyso-lipids + stearoyl-CoA (*C*), different isomers of oleoyl-LPE + stearoyl-CoA (*D*), and different isomers of oleoyl-LPE + stearoyl-CoA or palmitoyl-LPC + oleoyl-CoA or oleoyl-glycerol + stearoyl-CoA (*E*). *F*, the acyl specificity was determined in *E. coli* membranes expressing MBP-LPGAT1 in the presence of 25 μM 1-lyso-2-oleoyl-PE and 25 μM of each acyl-CoA species. *G* and *H*, acyltransferase activities were measured in liver microsomes from *Lpgat1*^*+/+*^ or *Lpgat1*^*−/−*^ mice. Substrates included the indicated lysolipids and acyl-CoAs. The graphs show replicate measurements, mean values, and SEM. Comparisons were made by *t*-tests. LPA, lysophosphatidic acid; LPC, lysophosphatidylcholine; LPE, lysophosphatidylethanolamine; LPG, lysophosphatidylglycerol; LPGAT1, lysophosphatidylglycerol acyltransferase 1; LPI, lysophosphatidylinositol; LPS, lysophosphatidylserine; MG, monoglyceride; PE, phosphatidylethanolamine.

Because our data did not confirm any effect on PG, the purported product of LPGAT1, we re-examined the substrate specificity of the enzyme. First, we expressed His-tagged murine LPGAT1 in Sf9 insect cells and affinity-purified the recombinant protein. Surprisingly, the purified enzyme was more active with lysophosphatidylethanolamine (LPE) than with LPG. It showed also some activity with MG but only minimal activity with lysophosphatidic acid, lysophosphatidylcholine (LPC), lysophosphatidylinositol, and lysophosphatidylserine (Fig. 2*A*). However, the yield of LPGAT1 in insect cells was low and not very reproducible from experiment to experiment. In order to create a more robust enzyme preparation, we expressed murine LPGAT1 fused to maltose-binding protein (MBP) in *Escherichia coli*, a technique known to produce large amounts of recombinant protein. Indeed, we obtained ample MBP-LPGAT1 that could be solubilized in Triton X-100 and purified to near homogeneity on Amylose resin (Fig. 2*B*). However, we were unable to recover acyltransferase activity. In order to generate an active enzyme, we lowered the expression level, which tends to lessen denaturation of the recombinant protein. Furthermore, we harvested plasma membranes instead of total cells because we wanted to collect an enzyme that was integrated in the membrane. Indeed, the modified method produced *E. coli* membranes with an acyltransferase activity that was 170-fold higher than that of the control (expression of MBP alone). When we analyzed the substrate specificity, we again found a higher acyltransferase activity with LPE than with LPG or MG and no activity with lysophosphatidic acid and LPC (Fig. 2*C*).

Since our data showed that LPGAT1 affected palmitate and stearate, fatty acids typically esterified to the sn-1 position, we asked whether LPGAT1 has any regiospecificity. Therefore, we compared the activity of membrane-bound MBP-LPGAT1 with the isomeric substrates 1-oleoyl-2-lyso-PE and 1-lyso-2-oleoyl-PE. The enzyme had an about 4-fold higher activity with 1-lyso-2-oleoyl-PE, suggesting that LPGAT1 prefers to react with the sn-1 hydroxyl group (Fig. 2*D*). Other lipids, such as LPC and MG, also elicited a higher activity with the 2-acyl isomer than the 1-acyl isomer, but their activities were negligible compared to the activity with 1-lyso-2-acyl-PE (Fig. 2*E*). Next, we determined the acyl specificity of MBP-LPGAT1 by comparing the substrates palmitoyl-CoA, stearoyl-CoA, linoleoyl-CoA, and arachidonoyl-CoA. The assay demonstrated a clear preference for saturated acyl-CoAs and a substantially higher activity with stearoyl-CoA than with palmitoyl-CoA (Fig. 2*F*).

To verify the function of LPGAT1, we determined the effect of LPGAT1 deletion on the endogenous acyltransferase activity in liver microsomes. Surprisingly, we found that WT microsomes had an about 4-fold higher acyltransferase activity with 1-lyso-2-oleoyl-PE than with 1-oleoyl-2-lyso-PE. Deletion of LPGAT1 eliminated any activity with either substrate, suggesting that LPGAT1 is the principal endogenous stearoyl-CoA:LPE acyltransferase of liver microsomes (Fig. 2*G*). Although LPGAT1 deletion abolished all stearoyl-CoA:LPE acyltransferase activity, it had little effect on linoleoyl-CoA:LPE acyltransferase activity, confirming the stearoyl specificity of the enzyme (Fig. 2*H*). Finally, we determined the effect of LPGAT1 deletion on acyltransferase activities with LPG, MG, and LPC. We found that normal microsomes reacylated 1-oleoyl-2-lyso-PG and 2-oleoyl-glycerol at a much lower velocity than LPE. Both activities were only mildly affected by LPGAT1 deletion. Also, reacylation of palmitoyl-LPC, measured with oleoyl-CoA to avoid the formation of poorly soluble disaturated PC, was not affected by LPGAT1 deletion regardless of whether 1-palmitoyl-2-lyso-PC or 1-lyso-2-palmitoyl-PC was the substrate (Fig. 2*H*).

In summary, our data demonstrate that LPGAT1 is an LPE acyltransferase with strong preference for stearoyl-CoA and the sn-1 hydroxyl group. Importantly, it accounts for most of the LPE acyltransferase activity of liver microsomes.

### Lysophosphatidylglycerol acyltransferase 1 deletion inhibits the *de novo* synthesis of lipids in hepatocytes

The substrate specificity of LPGAT1 and the effect of LPGAT1 deletion on the murine lipidome suggest that the enzyme participates in the remodeling of saturated fatty acids at the sn-1 position of PE with secondary consequences for the products of the PE methylation pathway. To verify this conclusion, we determined the effect of LPGAT1 deletion on the lipid fluxome by global ^13^C flux analysis. We incubated primary hepatocytes with ^13^C_6_-glucose and monitored the formation of ^13^C-isotopomers by quantitative mass spectrometry. We have previously shown that the relative abundancies of discrete isotopomers can be used to calculate the rate of *de novo* synthesis of the glycerol backbone of different lipid species (19).

**Figure 3:**
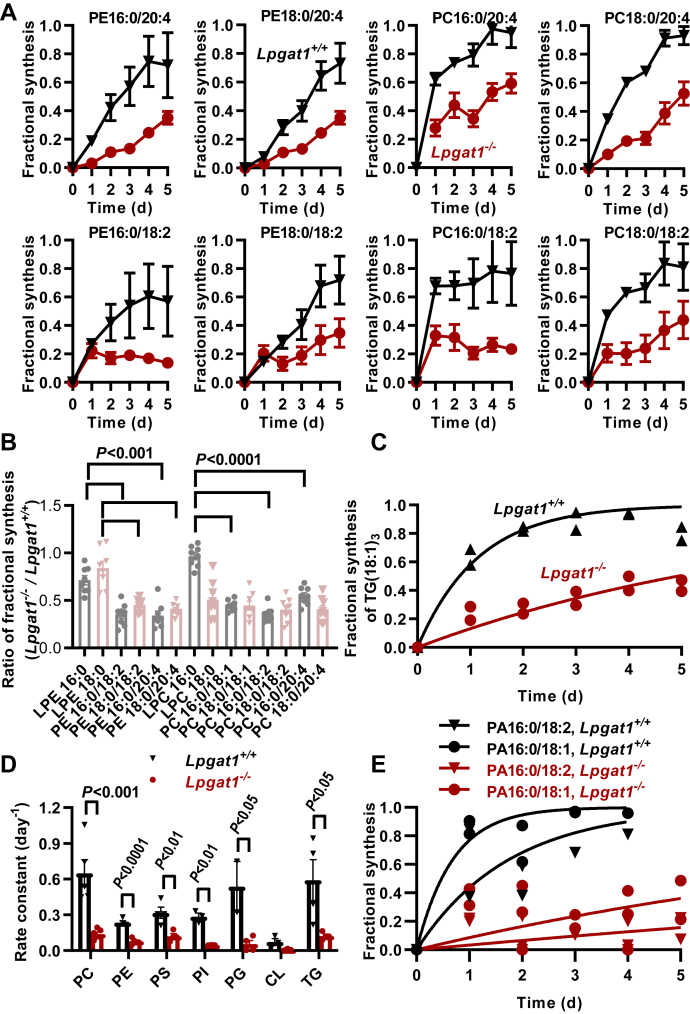
**Lysophosphatidylglycerol acyltransferase 1 deletion inhibits lipid synthesis in primary hepatocytes.** Hepatocytes were isolated from the livers of *Lpgat1*^*+/+*^ and *Lpgat1*^*−/−*^ mice and cultured in the presence of ^13^C_6_-glucose for 5 days. The cells were harvested at different time points and lipids were analyzed by LC-MS/MS. Fractional syntheses of lipid species were determined by isotopomer distribution analysis. Rate constants of abundant molecular species were determined by nonlinear regression of the time dependence of fractional syntheses. *A*, fractional syntheses of different PE and PC species. The data are means with range (N = 2). *B*, ratio of fractional syntheses (*Lpgat1*^*−/−*^*/Lpgat1*^*+/+*^) of different PE and PC species. Comparisons were made by t-tests. *C*, fractional synthesis of trioleoyl-glycerol. *D*, rate constants of different phospholipid classes. Each point represents a separate molecular species. The data are means ± SEM. Comparisons were made by t-tests. *E*, fractional syntheses of different PA species. CL, cardiolipin; LPC, lysophosphatidylcholine; LPE, lysophosphatidylethanolamine; PA, phosphatidic acid; PC, phosphatidylcholine; PE, phosphatidylethanolamine; PG, phosphatidylglycerol; PI, phosphatidylinositol; PS, phosphatidylserine; TG, triglyceride.

As expected, we found that LPGAT1 deletion sharply reduced the rate of synthesis of 1-stearoyl-2 acyl-PE species, such as PE18:0/20:4 and PE18:0/18:2. However, the effect was not specific to 1-stearoyl-2 acyl-PE as the synthesis of the corresponding 1-palmitoyl-2-acyl-PE species (PE16:0/20:4, PE16:0/18:2) decreased as well and so did the synthesis of all matching PC species, including PC18:0/20:4, PC18:0/18:2, PC16:0/20:4, and PC16:0/18:2 (Fig. 3*A*). These data hinted at a wider effect of LPGAT1 on lipid biosynthesis. While the synthesis of PE and PC decreased about equally, the lysophospholipids LPE16:0, LPE18:0, and LPC16:0, which are mostly 1-acyl-2-lyso intermediates of the sn-2 remodeling pathway, were not affected (Fig. 3*B*). These data are consistent with the notion that LPGAT1 is not involved in the Lands cycle.

Further analysis of the fluxome demonstrated that LPGAT1 deletion reduced the *de novo* synthesis of TG (Fig. 3*C*) and the *de novo* synthesis of phosphatidylserine, PI, and PG (Fig. 3*D*). Since LPGAT1 deletion had such an extensive effect on lipid biosynthesis, we wondered whether it affected the common first step, which is the formation of PA. Although the endogenous concentration of PA species is very low, we were able to identify the two main products of glycerol-3-phosphate acylation, PA16:0/18:1 and PA16:0/18:2 (20), and their corresponding ^13^C_3_ isotopomers in our mass spectra. Analysis of these species showed that LPGAT1 deletion reduced the *de novo* synthesis of PA in hepatocytes (Fig. 3*E*). However, LPGAT1 deletion did not affect PA formation in isolated liver microsomes supplied with glycerol-3-phosphate and palmitoyl-CoA plus stearoyl-CoA (Fig. S6). This suggested that although PA formation was reduced in *Lpgat1*^*−/−*^ hepatocytes, the expression of the rate-limiting glycerol-3-phosphate acyltransferase remained normal. It also showed that LPGAT1 was not directly involved in any of the acyl transfer reactions that converted glycerol-3-phosphate into PA.

In summary, our data indicate that LPGAT1 deletion induced the inhibition of global lipid biosynthesis in hepatocytes. While the mechanism of this phenomenon remains to be identified, the results suggest a central role of LPGAT1 in the cellular control of lipid metabolism.

## Discussion

Palmitate and stearate are the two principal saturated fatty acids linked to the sn-1 carbon atoms of mammalian phospholipids. Since phospholipid species formed by *de novo* synthesis contain more palmitate and less stearate than endogenous species (6, 20), the palmitate/stearate ratio has to be adjusted during postsynthetic processing. The relative abundance of palmitate and stearate seems to be significant. Studies have demonstrated that either the genetic manipulation of the palmitate/stearate ratio in phospholipids (21) or the dietary alteration of the palmitate/stearate ratio (22) affect insulin resistance and body fat ratio. Thus, it seems likely that the palmitate/stearate ratio requires regulation. Already more than 30 years ago, it has been postulated that palmitoyl species can be converted into stearoyl species by sn-1 remodeling but the enzyme catalyzing this process has remained elusive (6).

**Figure 4:**
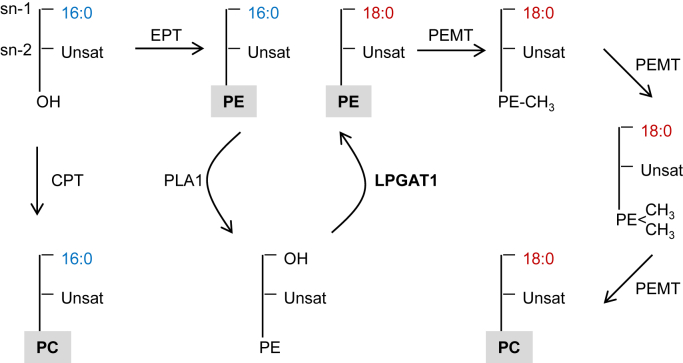
**Proposed role of LPGAT1 in phospholipid metabolism.** Lysophosphatidylglycerol acyltransferase 1 is involved in sn-1 remodeling of PE, which alters the palmitate-stearate balance of PE in favor of stearate. Phosphatidylethanolamine species formed by LPGAT1 are methylated to PC, which increases the stearate content of PC. 16:0, palmitate; 18:0, stearate; CPT, cholinephosphotransferase; EPT, ethanolaminephosphotransferase; LPGAT1, lysophosphatidylglycerol acyltransferase 1; PC, phosphatidylcholine; PE, phosphatidylethanolamine; PEMT, PE methyltransferase; PLA1, phospholipase A1; Unsat, unsaturated fatty acid.

In this paper, we identify LPGAT1 as the acyltransferase required for the postulated sn-1 remodeling and hypothesize that it works in tandem with a yet to be identified phospholipase A1 (Fig. 4). We show that LPGAT1 is an sn-1 specific acyl-CoA:LPE acyltransferase with a >10-fold preference for saturated fatty acids over unsaturated fatty acids and a 2-fold preference for stearoyl-CoA over palmitoyl-CoA. Consistent with the preference for stearoyl-CoA, we demonstrate that genetic ablation of LPGAT1 results in a substantial increase in the palmitate/stearate ratio in PE. The notion that LPGAT1 acts in concert with a phospholipase A1 is supported by the accumulation of unsaturated LPE species (presumably 1-lyso-2-acyl-PEs) in *Lpgat1*^*−/−*^ mice (Table S1).

Our discovery raises a number of interesting questions. First, it raises the question whether a specific phospholipase A1 is involved in the postulated sn-1 remodeling or whether multiple phospholipases provide substrate to LPGAT1. Thus, a crucial next step will be to identify the phospholipase(s) that collaborate with LPGAT1. Second, the inhibition of global lipid biosynthesis in *Lpgat1*^*−/−*^ mice is surprising and raises the question of how PE remodeling controls phospholipid biosynthesis. New experiments will be necessary to determine whether LPGAT1 affects lipid fluxes through the regulation of enzyme expression or posttranslational modification or whether the products of LPGAT1 (stearoyl-PEs) have an effect on lipid fluxes. Also, it has to be investigated whether downstream pathways like PE methylation or lipoprotein assembly have any role in the regulation of lipid fluxes.

Since LPGAT1 KO increased the palmitate/stearate ratio of PC, yet LPGAT1 did not have any intrinsic LPC acyltransferase activity, we suspect that LPGAT1-remodeled PE is converted to PC by the PE methylation pathway. In support of this notion, we found that LPGAT1 deletion had the same effect on the methylation pathway intermediate dMePE as it had on PE and PC. It remains unclear how LPGAT1 is linked to the PE methylation pathway except that both LPGAT1 (14) and PEMT (23) are residents of the mitochondria-associated endoplasmic reticulum. The fact that PC is as much affected as PE by LPGAT1 even though PE methylation contributes only about 30% of the total PC synthesis (18) strongly suggests that LPGAT1-remodeled PE species (*i.e.*, stearoyl-PEs) are selectively methylated to PC. The idea of an LPGAT1-PEMT link is consistent with data demonstrating a higher proportion of 1-stearoyl-2-polyunsaturated species in PEMT-derived PC compared to PC formed by the Kennedy pathway (24). Thus, LPGAT1 is involved in balancing the two principal saturated fatty acids, palmitate and stearate, in the two most abundant phospholipids, PE and PC (Fig. 4).

Involvement of LPGAT1 in lipoprotein metabolism has been suggested because LPGAT1 deficiency caused a decrease of TG and cholesterol in serum but an increase of TG and cholesterol in liver (14, 17). Our data show that LPGAT1 deficiency decreases TG synthesis, which in theory may reduce the availability of TG for lipoprotein assembly and therefore reduce the serum concentration of TG. However, our results do not support the idea that LPGAT1 is directly involved in TG synthesis *via* acyl transfer to MG, as proposed by others (17). We found that the acyltransferase activity of LPGAT1 with MG as substrate was only about 3% of its corresponding activity with LPE as substrate. Thus, acyl-CoA:MG acyltransferase activity is unlikely an inherent function of LPGAT1.

Instead, our work points to two alternative explanations for the involvement of LPGAT1 in lipoprotein metabolism. First, our data suggest that LPGAT1 provides substrates to the PE methylation pathway, which is known to be involved in hepatic lipoprotein secretion (25, 26, 27). It appears that different PC pools, derived from both the PE methylation pathway and the Kennedy pathway, are necessary for normal lipoprotein assembly (18). Therefore, LPGAT1 may provide substrates to the PE methylation pathway that then produces PC, which in turn is required for lipoprotein assembly. Second, our data suggest that LPGAT1 is regulating lipid *de novo* synthesis, which could affect the formation of TG and, by extension, the assembly of lipoproteins. More work is necessary to establish whether any of these explanations is accurate.

The misidentification of LPGAT1 as LPG-specific acyltransferase, which led to its current name (12), can be attributed in part to the limited availability of substrates at the time when the enzyme was discovered and in particular to the lack of positional isomers. Also, the low expression level of the enzyme in insect cells made it difficult to distinguish the heterologous activity from endogenous activities (12). In contrast, we expressed LPGAT1 in *E. coli* membranes, achieving high enzymatic activities, and tested a wide range of substrates. By employing comprehensive quantitative mass spectrometry and by confirming the results in a KO mouse, we were able to demonstrate unequivocally that LPGAT1 is an sn-1 specific LPE acyltransferase.

In summary, we identified LPGAT1 as stearoyl-CoA:1-lyso-2-acyl-PE acyltransferase and provided preliminary evidence for its biological function. Lysophosphatidylglycerol acyltransferase 1 controls the composition of the saturated fatty acids linked to the sn-1 position of PE and the products of the PE methylation pathway and it has a regulatory effect on cellular lipid biosynthesis. Importantly, LPGAT1 deficiency has consequences for body fat content and life span. Together, our data identify LPGAT1 as a central regulatory enzyme, whose function is (i) to balance saturated fatty acids in the sn-1 position of PE and PC and (ii) to control lipid biosynthesis in the endoplasmic reticulum.

## Experimental procedures

### Mice

All protocols were approved by the Institutional Animal Care and Use Committee of the NYU School of Medicine and conform to the Guide for the Care and Use of Laboratory Animals published by the National Institutes of Health. Mice were housed in a temperature-controlled facility under a 12-h light/dark cycle with free access to drinking water and food. The LPGAT1 KO mouse strain (C57BL/6NJ-Lpgat1em1(IMPC)J/Mmjax, MMRRC stock #42167) was purchased from The Jackson Laboratory as a heterozygous breeding pair. Homozygous *Lpgat1*^*−/−*^ mice were identified by standard genotyping of clipped tails. The mice were fed a standard Chow diet. Lean body composition and fat mass were analyzed using a Lunar PIXIMus mouse DEXA densitometer (Lunar Corp). Prior to each use, the machine was calibrated using the Quality Control function with the phantom mouse provided by the manufacturer. The mice were imaged *in vivo* while anesthetized with isoflurane (induction, 2–3% mixed with medical oxygen at 1 l/min flow rates; maintenance 1%) delivered *via* nose cone. The mice were placed in the prone position and the paws taped in splayed position onto the plastic tray as recommended. The head was placed outside of the imaging area to yield consistent, sub-cranial body compositions (28).

### Lysophosphatidylglycerol acyltransferase 1 expression in human and mouse tissue

Human tissue RNAs were purchased from Amsbio LLC and stored at −80 °C until use. Mouse organs were harvested after transcardial perfusion with normal saline. The organs were snap frozen in liquid nitrogen and kept at −80 °C until use. In order to extract RNA, tissue samples were homogenized in TRIZOL (Ambion, Life Technologies) using a Dounce tissue grinder. Total RNA was isolated from different organs using TRIZOL reagent. RNA concentrations were measured by the Nano Drop ND-2000 instrument (Thermo Scientific, #ND-2000). An aliquot of 2 μg of RNA was applied to synthesize first strand cDNA using the Applied Biosystems High-Capacity cDNA Reverse Transcription Kit (Thermo Scientific, #4368813). Synthesized cDNA was then analyzed by quantitative real-time PCR with the ABI Prism 7000HT Sequence Detection System and the SYBR green master mix (all supplied by Applied Biosystems, Thermo Fisher Scientific). Human LPGAT1 was transcribed with the forward primer 5′-GCA GCT GTT GGT CAC GAT AA-3′ and the reverse primer 5′-ACA CTG ATG ATG TGC CTC CA-3′. Mouse LPGAT1 was transcribed with the forward primer 5′-CAA CAG CTG CTG GTT CTC AA-3′ and the reverse primer 5′-CGT GCT ACA AGT GCC TTC AA-3′. The LPGAT1 expression was normalized to the expression of endogenous 18S RNA. Human 18S RNA was transcribed with the forward primer 5′-GAG GGA GCC TGA GAA ACG G-3′ and the reverse primer 5′-GTC GGG AGT GGG TAA TTT GC-3′. Mouse 18S RNA was transcribed with the forward primer 5′-GAT CCG AGG GCC TCA CTA AAC-3′ and the reverse primer 5′-AGT CCC TGC CCT TTG TAC ACA-3′. The data were analyzed by the 2^−ΔΔCT^ method (29).

### Lipidomics

Lipids were extracted from tissue samples (50–100 mg wet weight) into chloroform/methanol as described (30). In brief, the samples were suspended in methanol/chloroform (2:1) and incubated at 37 °C for 30 min to denature proteins. An aliquot of 25 μl of an internal standard mixture (SPLASH from Avanti Polar Lipids) was added. Chloroform and water were added, the samples were vortexed, and phase separation was achieved by centrifugation. The lower phase was collected, dried under nitrogen, and redissolved in 0.1 ml chloroform/methanol (1:1). Lipids were analyzed by liquid chromatography electrospray-ionization tandem mass spectrometry (LC-ESI-MS/MS) using a QExactive HF-X instrument coupled directly to a Vanquish UHPLC (Thermo Scientific). An aliquot of 7 μl was injected into a Restek Ultra C18 reversed-phase column (100 × 2.1 mm; particle size 3 μm) that was kept at a temperature of 50 °C. Chromatography was performed with solvents A and B at a flow rate of 0.15 ml/min. Solvent A contained 600 ml acetonitrile, 399 ml water, 1 ml formic acid, and 0.631 g ammonium formate. Solvent B contained 900 ml 2-propanol, 99 ml acetonitrile, 1 ml formic acid, and 0.631 g ammonium formate. The chromatographic run time was 40 min, changing the proportion of solvent B in a nonlinear gradient from 30 to 35% (0–2 min), from 35 to 67% (2–5 min), from 67 to 83% (5–8 min), from 83 to 91% (8–11 min), from 91 to 95% (11–14 min), from 95 to 97% (14–17 min), from 97 to 98% (17–20 min), from 98 to 100% (20–25 min), and from 100 to 30% (25–26 min). For the remainder of the run time, the proportion of solvent B stayed at 30% (26–40 min). The mass spectrometer was operated in negative or positive ion mode. The spray voltage was set to 4 kV and the capillary temperature was set to 350 °C. MS1 scans were acquired at a resolution of 120,000, an AGC target of 1e6, a maximal injection time of 65 ms, and a scan range of 300 to 2000 m/z. MS2 scans were acquired at a resolution of 30,000, an AGC target of 3e6, a maximal injection time of 75 ms, a loop count of 11, and an isolation window of 1.7 m/z. The normalized collision energy was set to 30 and the dynamic exclusion time to 13 s. For lipid identification and quantitation, the data were analyzed by the software LipidSearch 4.1 SP1 (Thermo Scientific). The general database was searched with a precursor tolerance of 2 ppm, a product tolerance of 0.2 Da, an intensity threshold of 1.0%, and an m-score threshold of 5. Diglyceride and TG were analyzed as [M+NH_4_] adducts in positive ion mode. Phosphatidylcholine was analyzed as [M+HCOO] adduct in negative ion mode and all other phospholipids as [M-H] ions in negative ion mode.

### Lipid synthesis in primary hepatocytes

To isolate hepatocytes, mice were anesthetized with isoflurane. The skin was disinfected with 70% ethanol and laparotomy was performed. A 22G cannula was inserted into the portal vein and the inferior vena cava was cut open. The liver was perfused through the portal vein with 50 ml prewarmed (37 °C) wash solution (HBSS) and with 50 ml Liver Digestion Medium containing collagenase (Gibco Cat. No. 17703-034). Liver tissue was carefully removed without gall bladder and placed into a sterile 50 ml tube containing 5 ml digestion medium. Liver tissue was dissected and gently agitated to release hepatocytes. An aliquot of 15 ml of cold wash medium, containing low glucose Dulbecco’s modified Eagle’s medium (DMEM), was added to the dissected liver and the preparation was filtered to remove debris and undigested tissue. Hepatocytes were collected by centrifugation and washed three times with 20 ml of cold wash medium. Washed hepatocytes were cultured in 10 ml low-glucose DMEM containing 10% fetal bovine serum (FBS). Nonadherent hepatocytes were removed after 1 h at 37 °C. Fresh medium was added and hepatocytes were cultured overnight. After overnight culture, the medium was replaced with labeling medium containing glucose-free DMEM, 2.5 g/l ^13^C_6_-glucose, and 10% FBS. The labeling medium was replaced every 24 h for up to 5 days. The cells were collected with a sterile cell scraper, spun at 200*g* for 5 min, and kept at −80 °C until ^13^C flux analysis, which was performed as described (19). Briefly, lipids were extracted and injected into the LC-ESI-MS/MS system specified above. Lipid species were identified by the software LipidSearch 4.1 SP1 (Thermo Scientific). ^13^C_3_ isotopomers had the same retention time as the monoisotopic ion and a mass that was 3.0101 Da larger than the monoisotopic mass. Isotopomers were quantified in Xcalibur 4.0 using a time window of 0.1 min centered at their peak retention time. Fractional syntheses were calculated from the relative abundances of ^13^C_0_ and ^13^C_3_ isotopomers at different time points. The labeling of the intracellular precursor glycerol-3-phosphate was determined from the ^13^C_0_/^13^C_3_/^13^C_6_ isotopomer pattern of PG (19). Rate constants were estimated by nonlinear regression to the time evolution of the fractional syntheses.

### Expression of His-tagged LPGAT1 in the Sf9-baculovirus system

Murine LPGAT1 cDNA was amplified with the forward primer 5′-AATAGAATTCATGCACCACCACCACCACCACGCCGTGACCGTGGAAG-3′ and the reverse primer 5′-AGTGCGGCCGCTAAAATAGACAATGGTAAAAATACTGAATGATGTGGTAC-3'. The product was inserted into the vector pVL1393, which was then used to transform DH5α competent *E. coli* cells. pVL1393-His-LPGAT1 DNA of positive clones was purified with the QIAGEN midi-prep kit. Aliquots of 0.1 μg of pVL1393-His-mLPGAT1 DNA and 0.5 μg of linearized baculovirus DNA (ProGreen, Cat. No. #A1, ABVector) were cotransfected by dropwise addition of the DNA emulsion with 10% Profectin (Cat. No. #T10, ABVector) to 60% confluent Sf9 cells. Cotransfections were performed in 1 ml serum-free and antibiotic-free TC-100 medium on a 6-well NUNC-delta plate. The cells were incubated at 27 °C for 24 h after which 1 ml 20% FBS-TC-100-1xPen-Strep medium was added up to a FBS concentration of 10%. The Sf9 cells were cultured for another 3 to 4 days at 27 °C. The recombinant baculovirus-containing supernatant was harvested and amplified by repeated baculovirus infections (2–3 cycles) to reach maximal protein expression. His-LPGAT1-expressing Sf9 cells were harvested by centrifugation at 1000 rpm for 5 min. The cells were homogenized, nuclei and debris were removed by centrifugation at 2000*g* for 10 min, and the membranes were isolated by centrifugation at 100,000*g* for 1 h (all at 4 °C). The membranes were solubilized in Triton X-100 (5 g/g protein) and His-tagged LPGAT1 was purified with NEBExpress Ni Resin (Cat. No. S1428S, New England BioLabs) following the manufacturer’s protocol. Protein concentrations were determined as described (31).

### Expression of MBP-tagged LPGAT1 in *E. coli*

Lysophosphatidylglycerol acyltransferase 1 cDNA was subcloned into the pMal-c2 vector using the Eco RI-Sal I/Xho I sites. *E. coli* BL21 competent cells were transformed with LPGAT1-pMal-c2 DNA. MBP-LPGAT1 protein expression was induced by adding IPTG to a final concentration of 0.9 mM. The cells were grown at 32 °C in an incubator-shaker operated at 250 rpm and harvested after 2.5 h at an A600 of 0.6 to 0.8. Cell pellets were resuspended in cold 0.25 M sucrose-0.25 mM EDTA-20 mM Tris pH 7.4 buffer supplemented with 1 mg/ml lysozyme-1× protease inhibitor (Cat. No. A32963, Pierce). The cells were sonicated for 2 to 3 min. Unbroken cells and debris were removed by centrifugation at 2000*g* for 20 min at 4 °C. *E. coli* membranes were collected by centrifugation at 100,000*g* for 1 h at 4 °C. As a control, we prepared *E. coli* membranes expressing MBP only by the same protocol. MBP-LPGAT1 was purified after solubilization in Triton X-100 (5 g/g protein) with Amylose resin (Cat. No. E8021S, New England BioLabs) following the manufacturer’s protocol. Protein concentrations were determined as described (31).

### Liver microsomes

Mice were anesthetized with isoflurane. The skin was disinfected and laparotomy was performed. The liver was excised and homogenized in cold isolation buffer (0.28 M sucrose, 10 mM Tris, pH 7.4, and 0.25 mM EDTA). All subsequent steps were carried out in refrigerated equipment. Debris, nuclei, and mitochondria were removed by consecutive spins at 1000*g* and 10,000*g* respectively. The post-10,000*g* supernatant was spun in an ultracentrifuge at 100,000*g* for 1 h. The 100,000*g* pellet was resuspended in isolation buffer, divided into aliquots, and stored at −80 °C. Protein concentration was determined as described (31).

### Enzyme activities

Lysophospholipid:acyl-CoA acyltransferase activities were assayed in 1 ml samples containing 10 mM Tris buffer (pH 7.4), 25 μM lysophospholipid or monoglyceride, and 50 μM acyl-CoA at 37 °C. Reactions were started by adding either purified LPGAT1 (1 μg protein), *E. coli* membranes expressing MBP or MBP-LPGAT1 (20 μg protein), or mouse liver microsomes (20 μg protein). The reactions were stopped after 2 or 5 min by adding 2 ml methanol and 1 ml chloroform. Lipids were extracted and analyzed by LC-ESI-MS/MS as described above. Intensities of products and internal standards were measured in Xcalibur 4.0. The glycerol-3-phosphate acyltransferase activity of liver microsomes was measured as described (32) in a medium containing 75 mM Tris (pH 7.5), 4 mM MgCl_2_, 1 mM mercaptoethanol, 5 mM glycerol-3-phosphate, 50 μM palmitoyl-CoA, and 50 μM stearoyl-CoA. The reaction was started by adding liver microsomes (0.1 mg protein) to 0.5 ml medium. The samples were incubated at 37 °C, and the reaction was stopped after 5 min by adding 2 ml methanol and 1 ml chloroform. Lipids were extracted and PA was measured by matrix-assisted laser-desorption-ionization time-of-flight mass spectrometry as described (33).

## Data availability

All data are contained within the article.

## Supporting information

This article contains supporting information.

## Conflict of interest

The authors declare that they have no conflicts of interest with the contents of this article.
