# Supplementary material for: LPGAT1 controls the stearate/palmitate ratio of phosphatidylethanolamine and phosphatidylcholine in sn-1 specific remodeling
Source: J Biol Chem. 2022 Feb 4;298(3):101685. doi: 10.1016/j.jbc.2022.101685 (PMC8892159; doi:10.1016/j.jbc.2022.101685)
Supplement: Supplemental Figure S1 [file mmc3.docx]

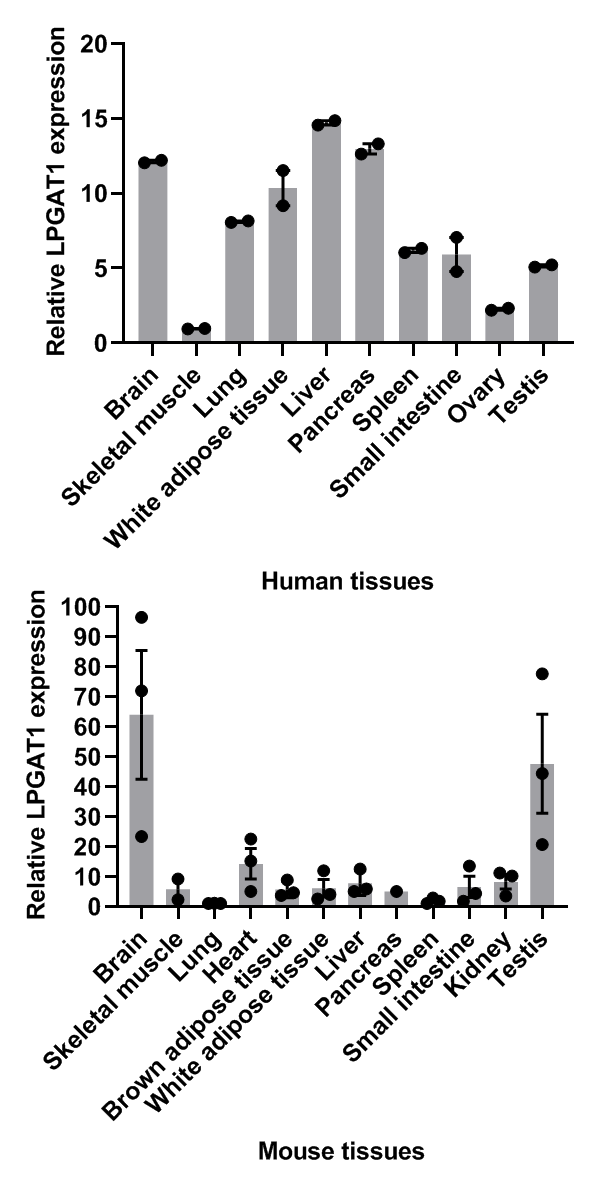


**Figure S1. LPGAT1 is ubiquitously expressed in human and mouse tissues.** The gene expression of LPGAT1 was analyzed by quantitative real-time PCR. Human tissues were obtained from a biorepository. Mouse tissues were harvested from 16 week-old C57BL/6 mice. Graphs show individual measurements in biological replicas, mean values, and ranges or SEM.
