# Supplementary material for: LPGAT1 controls the stearate/palmitate ratio of phosphatidylethanolamine and phosphatidylcholine in sn-1 specific remodeling
Source: J Biol Chem. 2022 Feb 4;298(3):101685. doi: 10.1016/j.jbc.2022.101685 (PMC8892159; doi:10.1016/j.jbc.2022.101685)
Supplement: Supplemental Figure S2 [file mmc4.docx]

**Figure S2. LPGAT1 deletion does not change the lipid class composition of liver.** Lipids were extracted from the livers of 5 months old mice and analyzed by LC-MS/MS. Data are means ± SEM (N=3). Abbreviations: Cer, ceramide; CL, cardiolipin; dMePE, dimethylphosphatidylethanolamine; FA, fatty acids; LdMePE, lysodimethylphosphatidylethanolamine; LPC, lysophosphatidylcholine; LPE, lysophosphatidylethanolamine; PA, phosphatidic acid; PC, phosphatidylcholine; PE, phosphatidylethanolamine; PEt, phosphatidylethanol; PG phosphatidylglycerol; PI, phosphatidylinositol; PIP; phosphatidylinositolphosphate; PMe, phosphatidylmethanol; PS, phosphatidylserine; SM, sphingomyelin.
