# Supplementary material for: LPGAT1 controls the stearate/palmitate ratio of phosphatidylethanolamine and phosphatidylcholine in sn-1 specific remodeling
Source: J Biol Chem. 2022 Feb 4;298(3):101685. doi: 10.1016/j.jbc.2022.101685 (PMC8892159; doi:10.1016/j.jbc.2022.101685)
Supplement: Supplemental Figure S3 [file mmc5.docx]

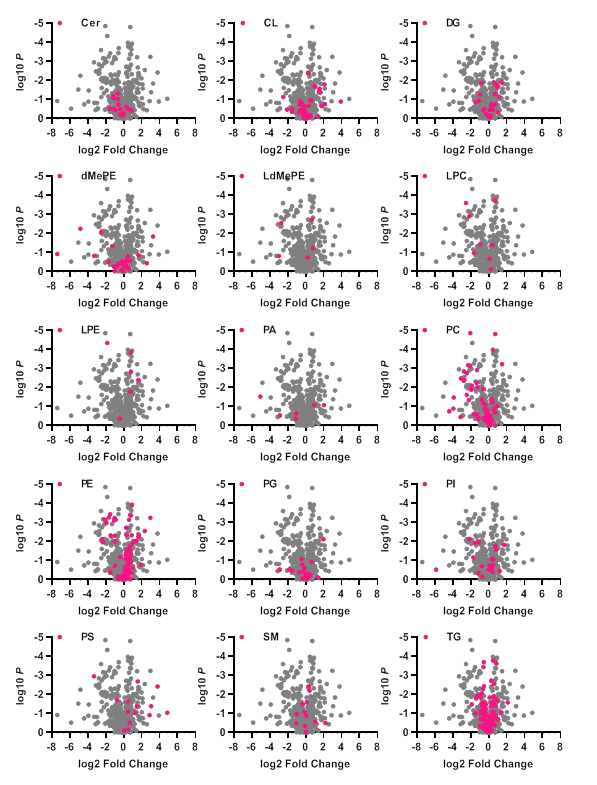


**Figure S3. Volcano plot identifies lipid species affected by LPGAT1 deletion.** Lipids were extracted from the livers of 5 months old mice and analyzed by LC-MS/MS. The lipidomes of *Lpgat1^+/+^* and *Lpgat1^-/-^* livers were compared by volcano plot. In each graph, species of the indicated lipid class are colored red.
