# Supplementary material for: LPGAT1 controls the stearate/palmitate ratio of phosphatidylethanolamine and phosphatidylcholine in sn-1 specific remodeling
Source: J Biol Chem. 2022 Feb 4;298(3):101685. doi: 10.1016/j.jbc.2022.101685 (PMC8892159; doi:10.1016/j.jbc.2022.101685)
Supplement: Supplemental Figure S4 [file mmc6.docx]

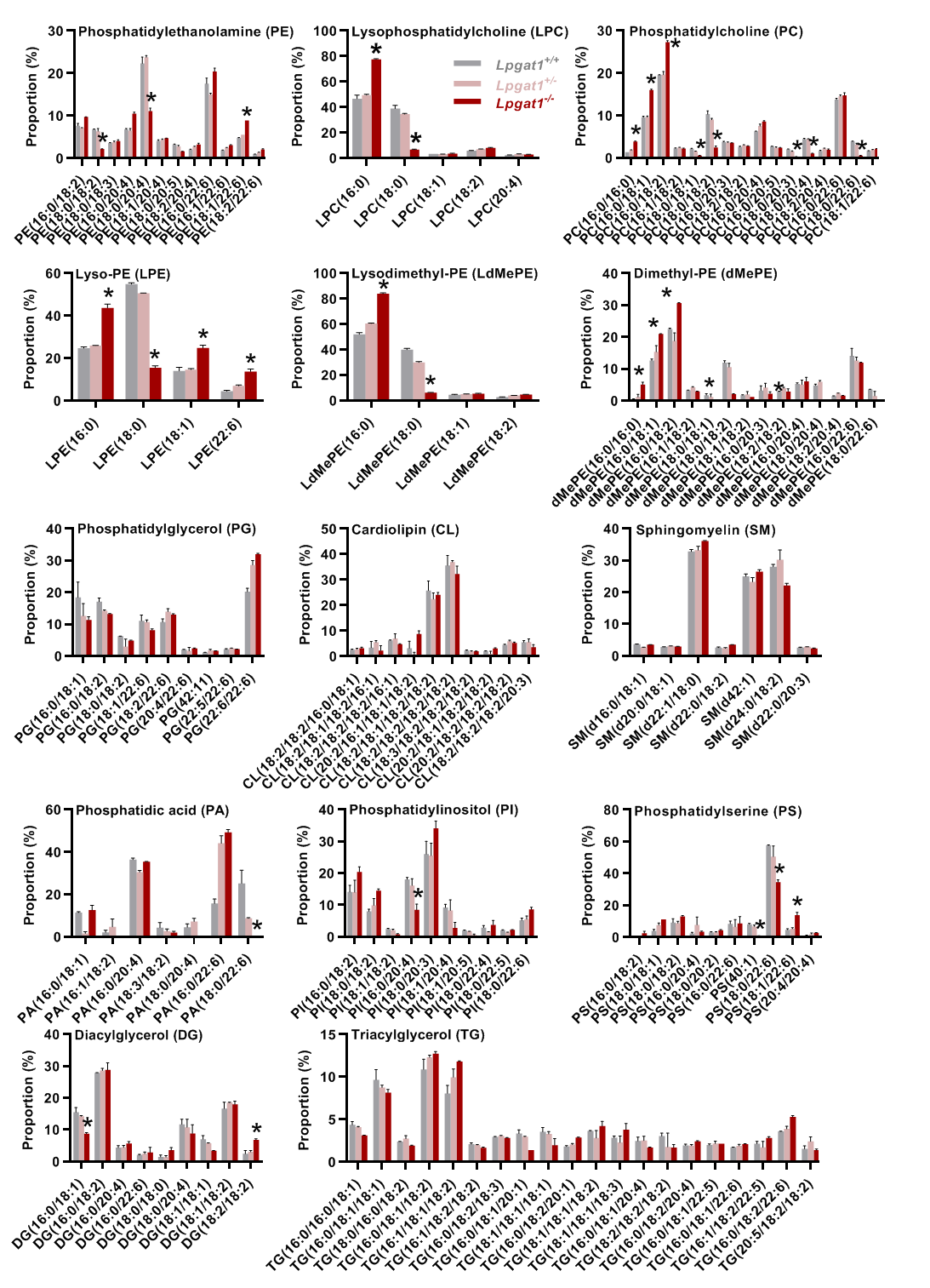


**Figure S4. LPGAT1 deletion increases the palmitate/stearate ratio of hepatic PE, dMePE, and PC.** Lipids were extracted from the livers of 5 months old mice and analyzed by LC-MS/MS. Graphs show the distribution of molecular species in different lipid classes. Only species with an abundance of >2% are listed. Data are means ± SEM (N=3). Asterisks mark species in which deletion of LPGAT1 caused a significant change in abundance (*Lpgat1^+/+^ vs Lpgat^-/-^*, *P*<0.05, t-test).
