# Supplementary material for: LPGAT1 controls the stearate/palmitate ratio of phosphatidylethanolamine and phosphatidylcholine in sn-1 specific remodeling
Source: J Biol Chem. 2022 Feb 4;298(3):101685. doi: 10.1016/j.jbc.2022.101685 (PMC8892159; doi:10.1016/j.jbc.2022.101685)
Supplement: Supplemental Figure S6 [file mmc8.docx]

**Figure S6. LPGAT1 deletion does not affect GPAT activity of mouse liver microsomes.** GPAT activity was measured in a medium containing 75 mM Tris (pH 7.5), 4 mM MgCl_2_, 1 mM mercaptoethanol, 5 mM glycerol-3-phosphate, 50 µM palmitoyl-CoA, and 50 µM stearoyl-CoA. The reaction was started by adding liver microsomes (0.1 mg protein) to 0.5 mL medium. Samples were incubated at 37°C and the reaction was stopped after 5 minutes by adding 2 mL methanol and 1 mL chloroform. Lipids were extracted and PA was measured by matrix-assisted laser-desorption-ionization time-of-flight MS. The graph shows replicate measurements, mean values, and SEM (N=6).
